# Supplementary material for: Reconstructing unseen transmission events to infer dengue dynamics from viral sequences
Source: Nat Commun. 2021 Mar 22;12:1810. doi: 10.1038/s41467-021-21888-9 (PMC7985522; doi:10.1038/s41467-021-21888-9)
Supplement: Supplementary file 1 — Supplementary Information [file 41467_2021_21888_MOESM1_ESM.pdf]

Reconstructing unseen transmission events to infer dengue dynamics from viral sequences

Supplementary information

Supplementary Figures 1-17

Supplementary Figure 1

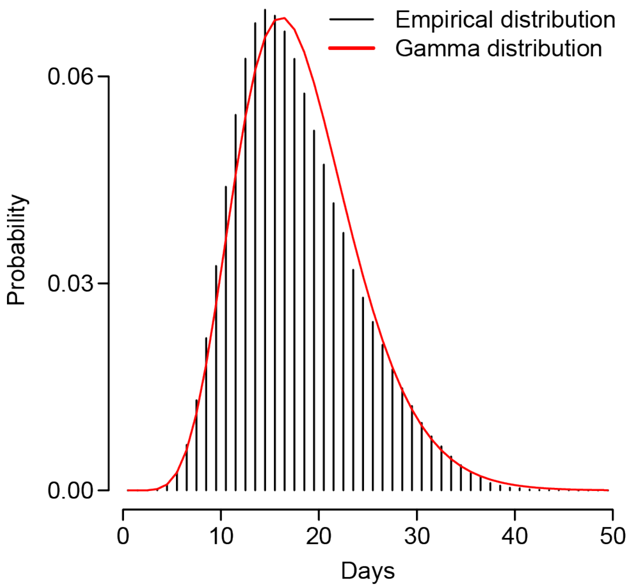

Supplementary Figure 1: Generation time distribution for dengue. We compare the empirical distribution function (black) with that using a gamma distribution (red).

Supplementary Figure 2

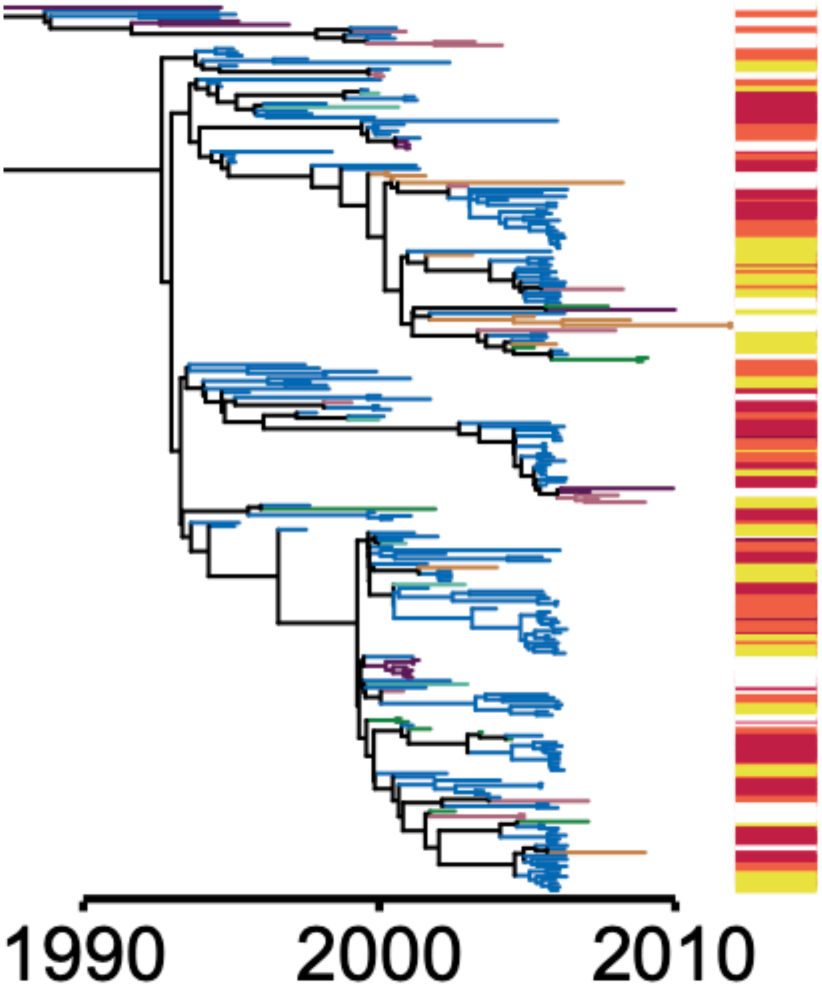

Supplementary Figure 2. Enlarged DENV1 Tree from Figure 1. The legend for the colours is the same as Figure 1.

Supplementary Figure 3

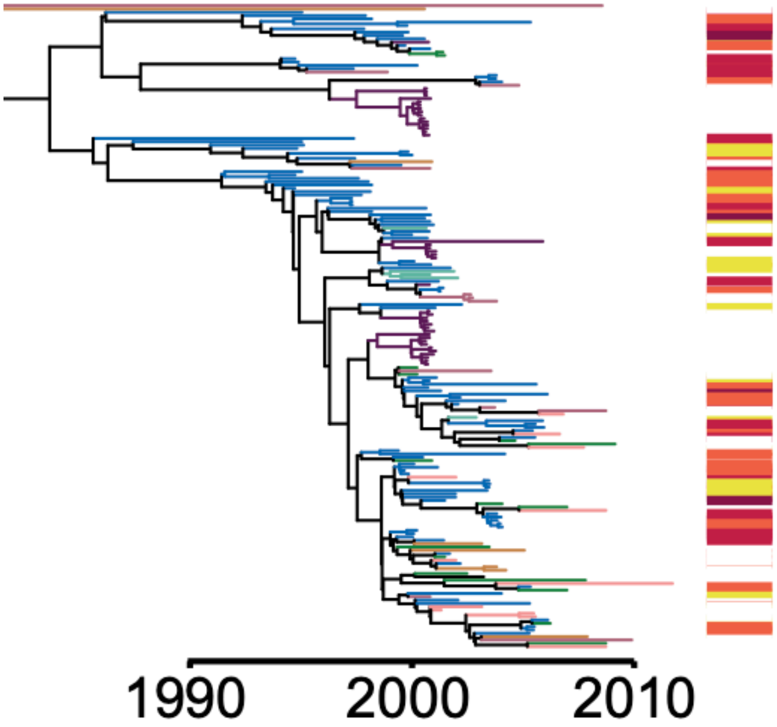

Supplementary Figure 3. Enlarged DENV2 Tree from Figure 1. The legend for the colours is the same as Figure 1.

Supplementary Figure 4

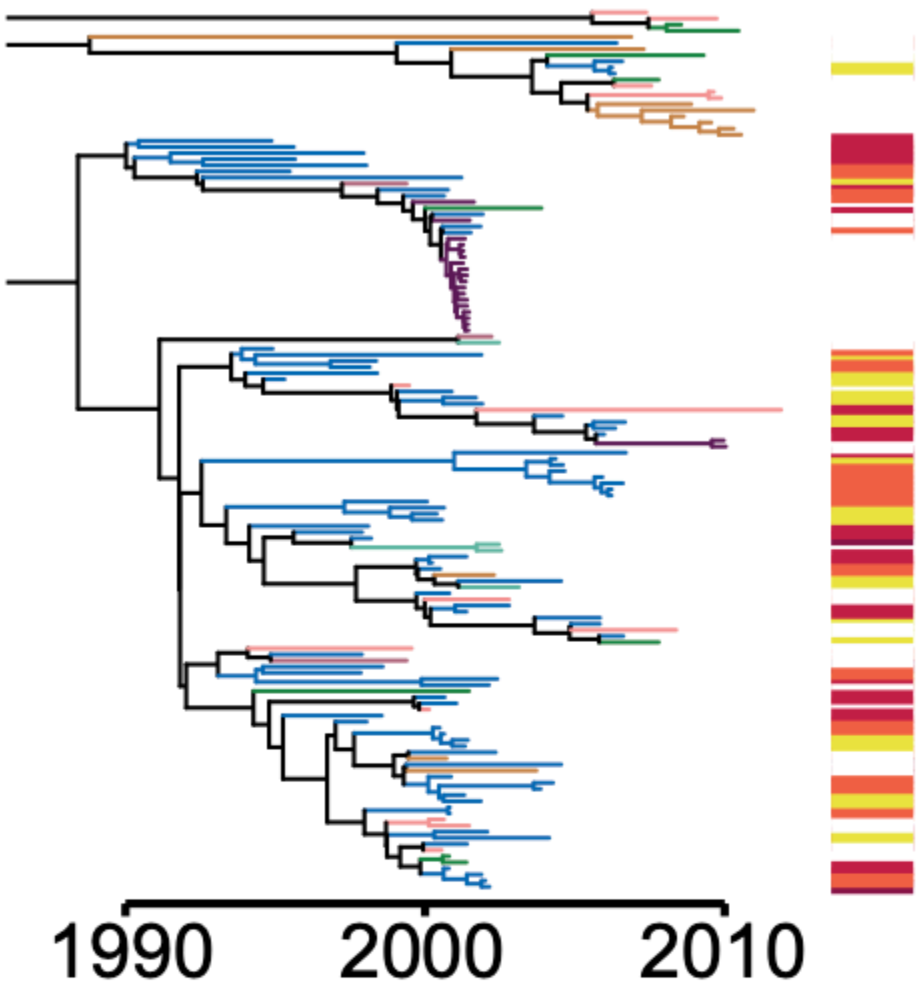

Supplementary Figure 4. Enlarged DENV3 Tree from Figure 1. The legend for the colours is the same as Figure 1.

Supplementary Figure 5

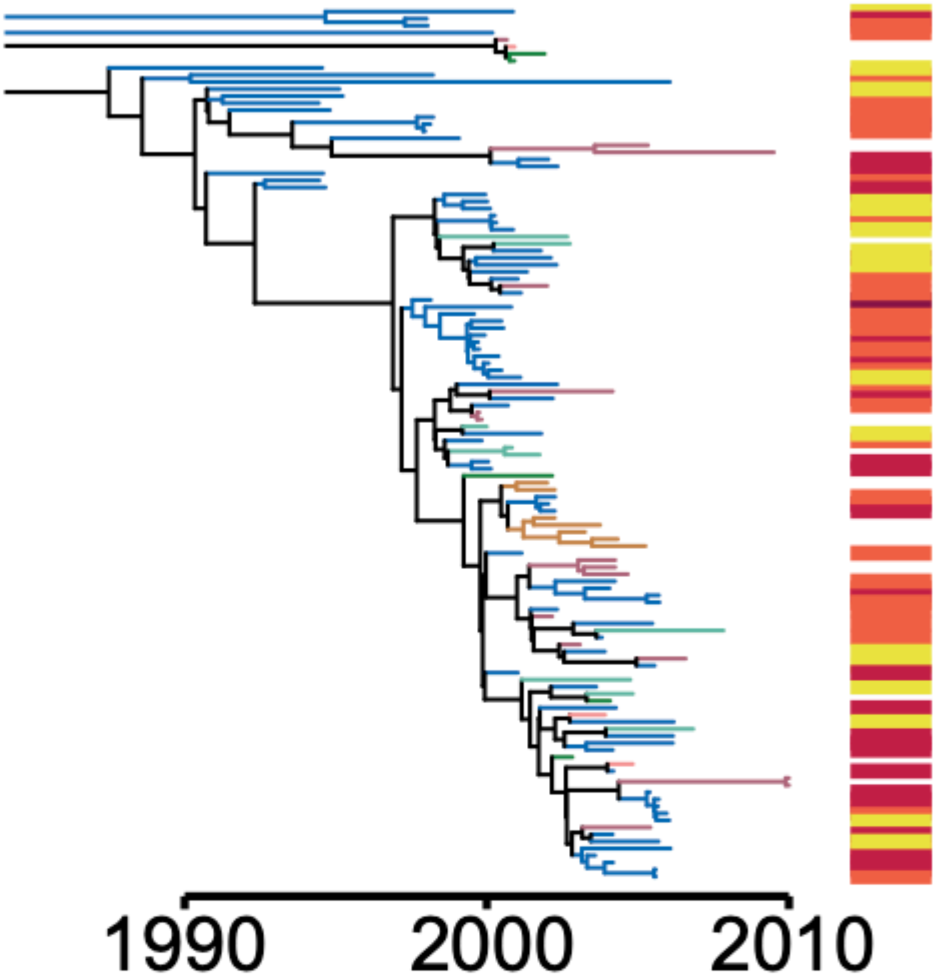

Supplementary Figure 5. Enlarged DENV4 Tree from Figure 1. The legend for the colours is the same as Figure 1.

Supplementary Figure 6

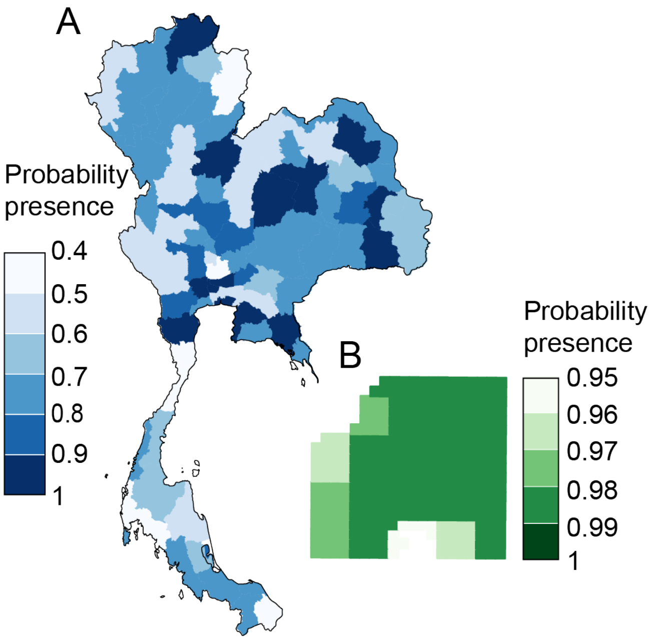

Supplementary Figure 6: Aedes aegypti maps. (A) Mean estimated probability of Aedes presence across each province in Thailand. **(B)** Mean estimated probability of Aedes presence across each cell in central Bangkok. Note the different scales in the two locations.

Supplementary Figure 7

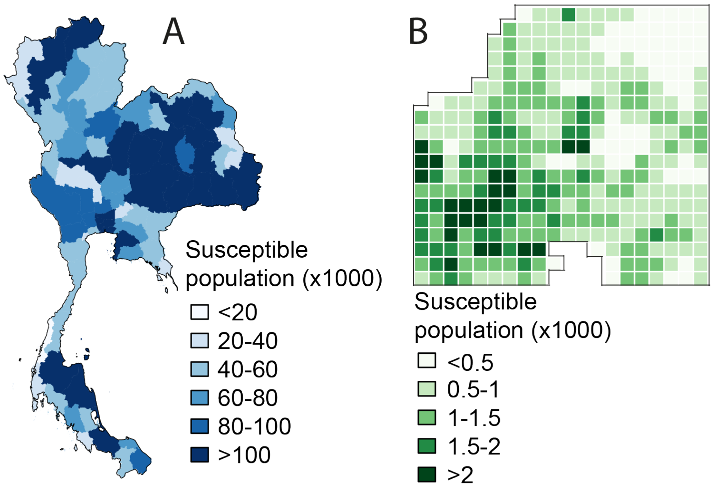

Supplementary Figure 7. Number of susceptible people. Average number of individuals that are either completely naive or have only been infected by one serotype when assuming an average force of infection of 0.04 per serotype in **(A)** each Thai Province and **(B)** in each 1km x 1km grid cell in Bangkok. Assumes the age structure of the population is approximately constant across Thailand.

Supplementary Figure 8

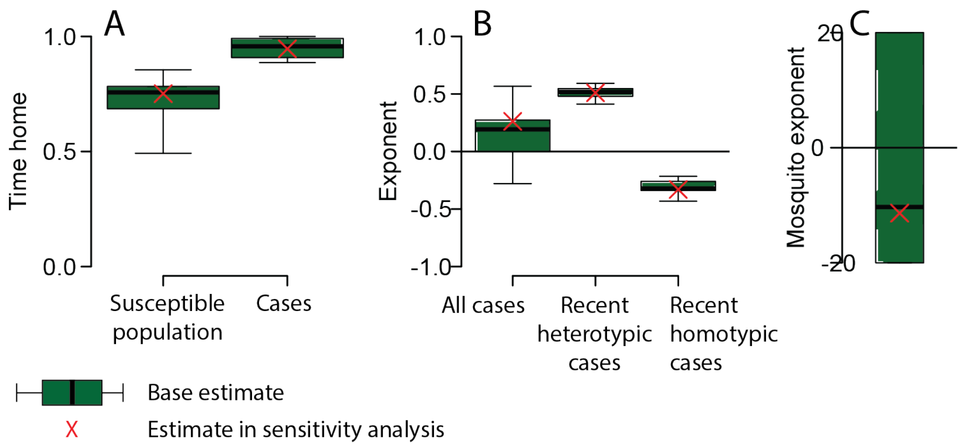

Supplementary Figure 8: Sensitivity analysis where assume location of MRCA is equal across all locations. We compare the inferences made when we assume that the probability the MRCA is in any location is proportional to the size of the population within that location (base model, green) with when we assume all locations have the same probability (red cross). **(A)** Estimates for the time in the home cell for the susceptible population and cases. **(B)** Exponents for all cases, recent homotypic cases and recent heterotypic cases and **(C)** **e**xponent for the mosquito suitability estimate. All uncertainty estimates are calculated from 100 bootstrap resamples. The boxplots represent the mean estimate, with the bounds of the box representing 25% and 75% quantiles and the minima and maxima representing 95% confidence intervals.

Supplementary Figure 9

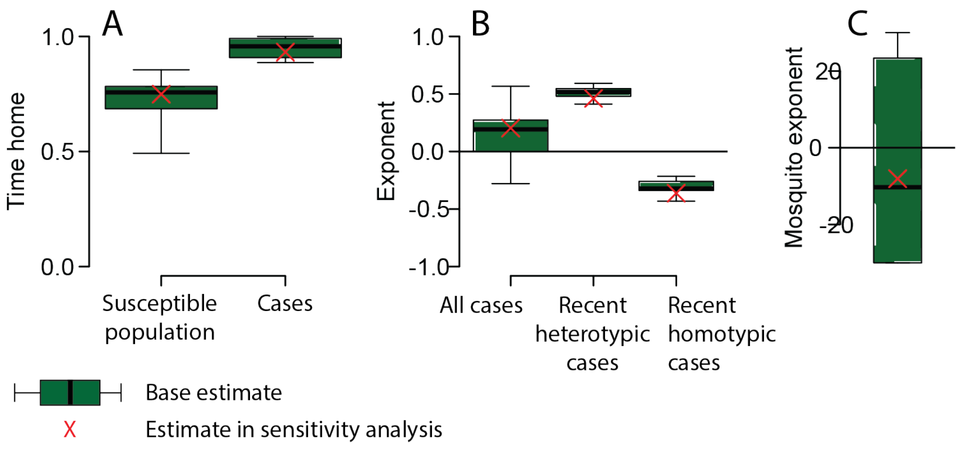

Supplementary Figure 9: Sensitivity analysis where inference based on 40 generations. We compare the inferences made when we consider pairs of sequences where the expected number of transmission generations between the MRCA and each tip is <25 generations (base model, green) with inferences when we use a maximum number of 40 transmission generation (red cross). **(A)** Estimates for the time in home cell for the susceptible population and cases. **(B)** Exponents for all cases, recent homotypic cases and recent heterotypic cases and **(C)** exponent for the mosquito suitability estimate. All uncertainty estimates are calculated from 100 bootstrap resamples. The boxplots represent the mean estimate, with the bounds of the box representing 25% and 75% quantiles and the minima and maxima representing 95% confidence intervals.

Supplementary Figure 10: SMILI Human Mobility data

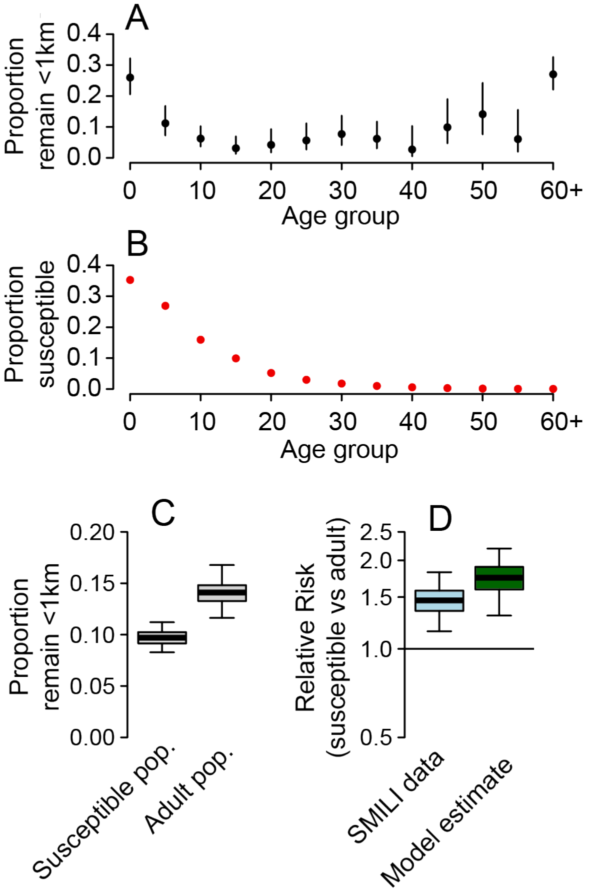

Supplementary Figure 10. Human mobility data from SMILI project (N=2011 individuals). (A) Proportion of individuals that reported having not travelled more than 1km within the last 7 days by 5 year age group with 95% confidence intervals. **(B)** Proportion of the population by five-year age group that is susceptible to infection (completely naive or monotypically immune) assuming a fixed force of infection of 0.04 per serotype/year. **(C)** Estimated average proportion of the susceptible population and adult population that has not travelled more than 1km in the last seven days. The boxplots represent the mean estimate, with the bounds of the box representing 25% and 75% quantiles and the minima and maxima representing 95% confidence intervals and are calculated from all 2011 individuals. **(D)** Relative risk of having not travelled more than 1km in the last seven days comparing the susceptible population with adults (blue) calculated from all 2011 SMILI participants and the relative risk of staying within a grid cell for susceptible individuals versus adults as inferred by the Bangkok model (green). The boxplots represent the mean estimate, with the bounds of the box representing 25% and 75% quantiles and the minima and maxima representing 95% confidence intervals.

Supplementary Figure 11

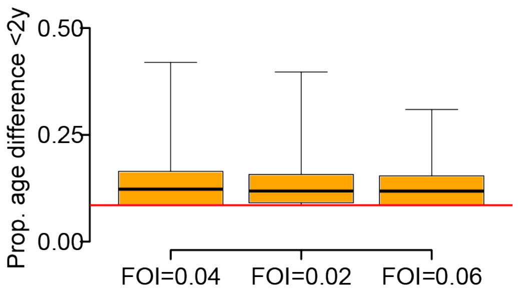

Supplementary Figure 11: Sensitivity analysis where assume different forces of infections. Estimates of the proportion of infections that are between individuals of within 2 years difference in age. The red line represents the scenario where there is no difference across all age groups and is the lowest possible value that can be taken. All uncertainty estimates are calculated from 100 bootstrap resamples. The boxplots represent the mean estimate, with the bounds of the box representing 25% and 75% quantiles and the minima and maxima representing 95% confidence intervals.

Supplementary Figure 12

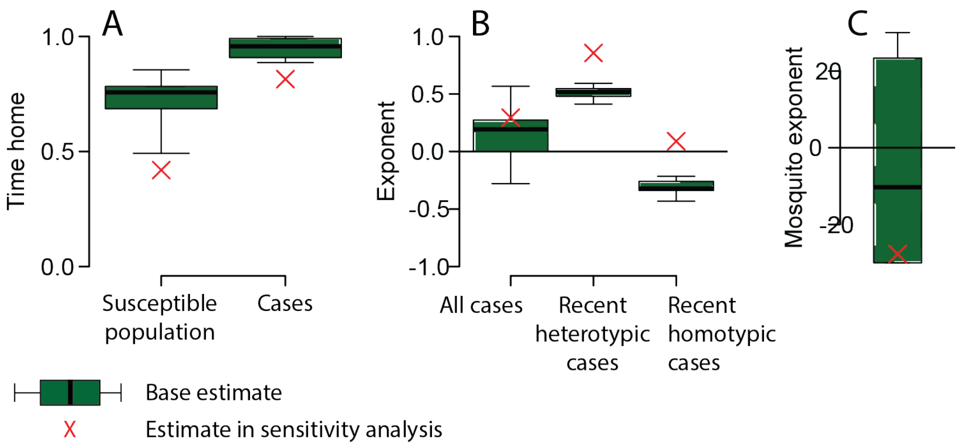

Supplementary Figure 12: Sensitivity analysis where assume unbiased observation. We compare the inferences made when we account for sampling probability (base model, green) with when we assume even sampling in space and time (red cross). **(A)** Estimates for the time spent in the home cell for the susceptible population and cases. **(B)** Exponents for all cases, recent homotypic cases and recent heterotypic cases and **(C) e**xponent for the mosquito suitability estimate. All uncertainty estimates are calculated from 100 bootstrap resamples. The boxplots represent the mean estimate, with the bounds of the box representing 25% and 75% quantiles and the minima and maxima representing 95% confidence intervals.

Supplementary Figure 13

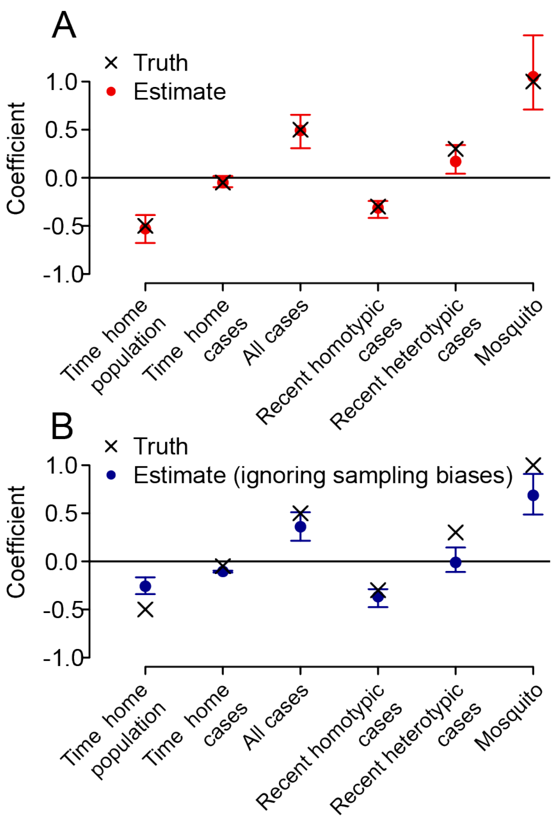

Supplementary Figure 13: Performance of model using simulated data with known parameter values. (A) Comparison of mean and 95% range of estimates (red) from 20 simulations with true values (black cross) when appropriately accounting for spatiotemporal biases in reporting. **(B)** Comparison of mean and 95% range of estimates (blue) from 20 simulations with true values (black cross) when assuming equal probability of reporting in all locations.

Supplementary Figure 14

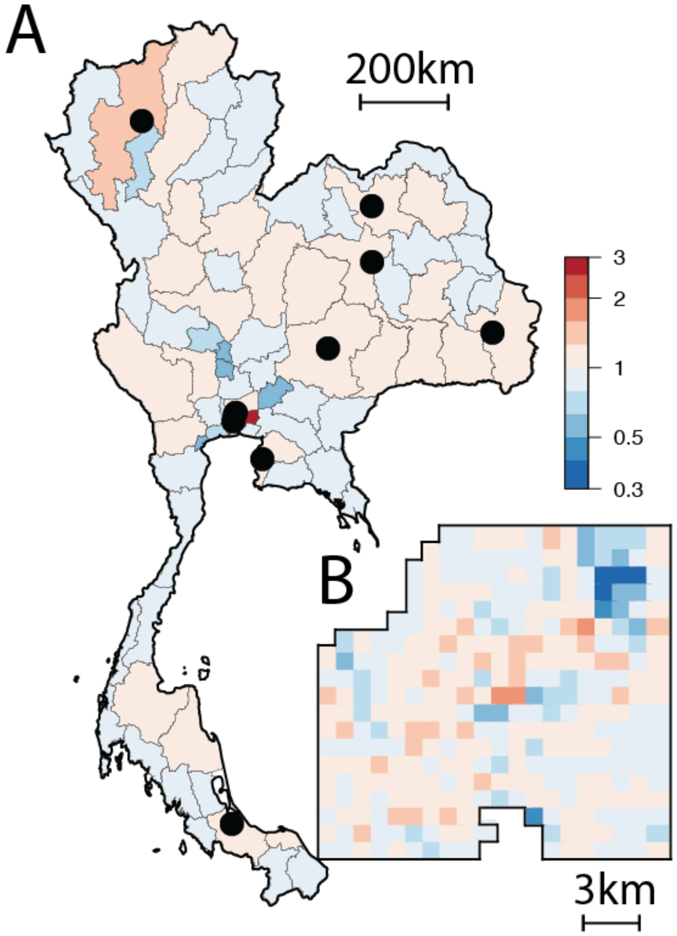

Supplementary Figure 14. (A) Relative risk of movement of virus to each province compared to moving to a randomly selected province in a single transmission generation. The black dots represent the 10 largest cities in Thailand. **(B)** Relative risk of movement of virus to each grid cell within central Bangkok after a single generation.

Supplementary Figure 15

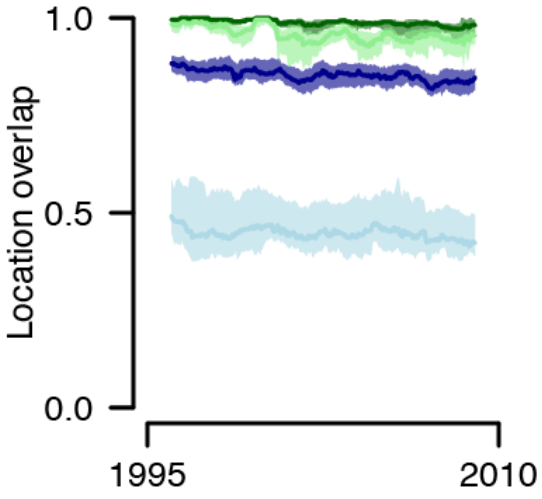

Supplementary Figure 15. Correlation in spread across serotypes. The probability of the likeliest non-home location being the same across serotypes in Bangkok and across provinces after a single transmission step (purple for Bangkok and dark green for provinces) and 20 generations (light blue for Bangkok and light green for provinces).

Supplementary Figure 16

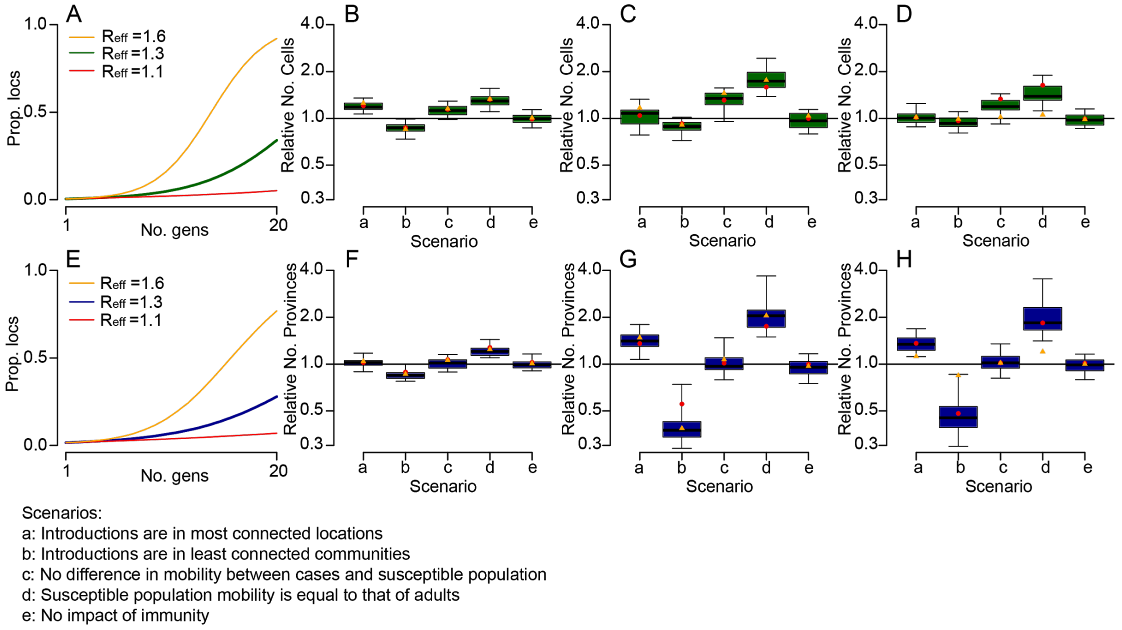

Supplementary Figure 16: Sensitivity analysis where assume different R_{eff} on forward simulations. (A) The proportion of locations within Bangkok that have at least one infection, comparing different R_{eff}. The number of Bangkok locations with at least one case for different scenarios relative to that in the base model after **(B)** one generation **(C)** 10 generations (approximately six months) and **(D)** 20 generations (approximately one year). **(E)** The proportion of Thai provinces that have at least one infection, comparing different R_{eff}. The number of Thai provinces with at least one case for different scenarios relative to that in the base model after **(F)** one generation **(G)** 10 generations (approximately six months) and **(H)** 20 generations (approximately one year). The red and orange dots in panels **(B)-(D)** and **(F)-(H)** represent the scenarios with R_{eff} of 1.1 and 1.6 respectively and the green/blue boxes represent the scenario with an R_{eff} of 1.3. The boxplots represent the mean estimate, with the bounds of the box representing 25% and 75% quantiles and the minima and maxima representing 95% confidence intervals. All uncertainty estimates are calculated from 100 repeated simulations.

Supplementary Figure 17

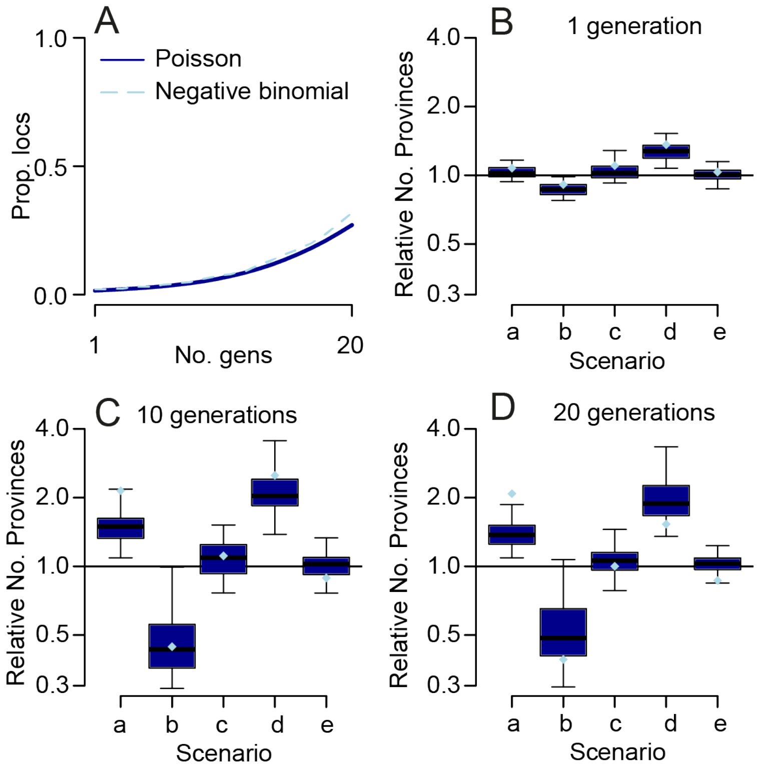

Scenarios:
a: Introductions are in most connected locations
b: Introductions are in least connected communities
c: No difference in mobility between cases and susceptible population
d: Susceptible population mobility is equal to that of adults
e: No impact of immunity

Supplementary Figure 17. Sensitivity analysis using reproduction numbers drawn from a negative binomial distribution. We repeat the analyses in S11 but instead the number of daughter infections is drawn from a negative binomial distribution with dispersion size of 1. Reff is fixed at 1.3 in both the models that use a Poisson distribution and the models that use a Negative Binomial distribution. All uncertainty estimates are calculated from 100 repeated simulations. The boxplots represent the mean estimate, with the bounds of the box representing 25% and 75% quantiles and the minima and maxima representing 95% confidence intervals.
