# Supplementary material for: Reconstructing unseen transmission events to infer dengue dynamics from viral sequences
Source: Nat Commun. 2021 Mar 22;12:1810. doi: 10.1038/s41467-021-21888-9 (PMC7985522; doi:10.1038/s41467-021-21888-9)
Supplement: Supplementary file 3 — Descriptions of Additional Supplementary Files [file 41467_2021_21888_MOESM3_ESM.docx]

Descriptions of Additional Supplementary Files

**Supplementary Data 1**

**Description:** GenBank Accession numbers for the dengue virus sequences used in the paper.
