# Supplementary material for: Reconstructing unseen transmission events to infer dengue dynamics from viral sequences
Source: Nat Commun. 2021 Mar 22;12:1810. doi: 10.1038/s41467-021-21888-9 (PMC7985522; doi:10.1038/s41467-021-21888-9)
Supplement: Supplementary file 5 — Reporting Summary [file 41467_2021_21888_MOESM5_ESM.pdf]

nature research

Corresponding author(s): Henrik Salje

Last updated by author(s): Sep 17, 2020

# Reporting Summary

Nature Research wishes to improve the reproducibility of the work that we publish. This form provides structure for consistency and transparency in reporting. For further information on Nature Research policies, see our Editorial Policies and the Editorial Policy Checklist.

## Statistics

For all statistical analyses, confirm that the following items are present in the figure legend, table legend, main text, or Methods section.

| n/a | Confirmed | |
|---|---|---|
| ☐ | ☒ | The exact sample size ($n$) for each experimental group/condition, given as a discrete number and unit of measurement |
| ☐ | ☒ | A statement on whether measurements were taken from distinct samples or whether the same sample was measured repeatedly |
| ☒ | ☐ | The statistical test(s) used AND whether they are one- or two-sided<br>*Only common tests should be described solely by name; describe more complex techniques in the Methods section.* |
| ☐ | ☒ | A description of all covariates tested |
| ☐ | ☒ | A description of any assumptions or corrections, such as tests of normality and adjustment for multiple comparisons |
| ☐ | ☒ | A full description of the statistical parameters including central tendency (e.g. means) or other basic estimates (e.g. regression coefficient) AND variation (e.g. standard deviation) or associated estimates of uncertainty (e.g. confidence intervals) |
| ☐ | ☒ | For null hypothesis testing, the test statistic (e.g. $F$, $t$, $r$) with confidence intervals, effect sizes, degrees of freedom and $P$ value noted<br>*Give P values as exact values whenever suitable.* |
| ☐ | ☒ | For Bayesian analysis, information on the choice of priors and Markov chain Monte Carlo settings |
| ☒ | ☐ | For hierarchical and complex designs, identification of the appropriate level for tests and full reporting of outcomes |
| ☒ | ☐ | Estimates of effect sizes (e.g. Cohen's $d$, Pearson's $r$), indicating how they were calculated |

*Our web collection on statistics for biologists contains articles on many of the points above.*

## Software and code

Policy information about availability of computer code

Data collection: Custom code was developed for the analyses. These has been made available via Zenodo. LandScan data (2010) is available from https://landscan.ornl.gov/landscan-datasets.

Data analysis: BEASTv1.10.4, R v4.0.3

For manuscripts utilizing custom algorithms or software that are central to the research but not yet described in published literature, software must be made available to editors and reviewers. We strongly encourage code deposition in a community repository (e.g. GitHub). See the Nature Research guidelines for submitting code & software for further information.

## Data

Policy information about availability of data

All manuscripts must include a data availability statement. This statement should provide the following information, where applicable:

- Accession codes, unique identifiers, or web links for publicly available datasets
- A list of figures that have associated raw data
- A description of any restrictions on data availability

All data is existing and used in a prior publication. For the data that is not already available, we will make publicly available on a GitHub server upon acceptance of the manuscript, with the exception of home cell coordinates for the Bangkok sequences due to identifiability concerns. All sequences are on GenBank.

# Field-specific reporting

Please select the one below that is the best fit for your research. If you are not sure, read the appropriate sections before making your selection.

☐ Life sciences ☐ Behavioural & social sciences ☒ Ecological, evolutionary & environmental sciences

For a reference copy of the document with all sections, see nature.com/documents/nr-reporting-summary-flat.pdf

# Ecological, evolutionary & environmental sciences study design

All studies must disclose on these points even when the disclosure is negative.

| | |
|---|---|
| Study description | Development of analytical methods to understand pathogen flows from sequence data - using dengue virus in Thailand as a case study |
| Research sample | All available dengue sequences from Thailand where we have spatial data. These are described in a previous publication (Salje et al., Science 2017). They consist of sequences obtained from individuals where the case home was also geocoded. |
| Sampling strategy | Comprehensive |
| Data collection | Existing data that was previously collected by investigators on this paper (Salje and Cummings). The home locations of individuals hospitalised with dengue were geocoded using base maps of the city. |
| Timing and spatial scale | Existing data from 1995 to 2012 at two spatial scales: within Bangkok - where the home 1km2 grid cell is known or at a Province level. The samples for sequencing were chosen at random and covered the widest range of dates available at the time where underlying address information was available. |
| Data exclusions | None |
| Reproducibility | We used a simulation framework to demonstrate we could recover parameters when they were known. This simulation framework was replicated 50 times with different sets of input parameters and the model was able to correctly identify these parameters each time. |
| Randomization | As all data was used in the analysis and we are not comparing different groups, no randomization was necessary |
| Blinding | There was no prospective data collection involved so blinding is not relevant to the study |

Did the study involve field work? ☐ Yes ☒ No

# Reporting for specific materials, systems and methods

We require information from authors about some types of materials, experimental systems and methods used in many studies. Here, indicate whether each material, system or method listed is relevant to your study. If you are not sure if a list item applies to your research, read the appropriate section before selecting a response.

## Materials & experimental systems

| n/a | Involved in the study | |
|---|---|---|
| ☒ | ☐ | Antibodies |
| ☒ | ☐ | Eukaryotic cell lines |
| ☒ | ☐ | Palaeontology and archaeology |
| ☒ | ☐ | Animals and other organisms |
| ☒ | ☐ | Human research participants |
| ☒ | ☐ | Clinical data |
| ☒ | ☐ | Dual use research of concern |

## Methods

| n/a | Involved in the study | |
|---|---|---|
| ☒ | ☐ | ChIP-seq |
| ☒ | ☐ | Flow cytometry |
| ☒ | ☐ | MRI-based neuroimaging |
